# Neoadjuvant chemotherapy with or without metformin in early-stage or locally advanced (non-metastatic) triple negative breast cancer—an open-label phase 2 randomized controlled trial

**DOI:** 10.3389/fonc.2025.1561692

**Published:** 2025-06-25

**Authors:** Vinod K. Ramani, Ajaikumar Basavalinga Sadashivaiah, Radheshyam Naik

**Affiliations:** ^1^ Preventive Oncology, Healthcare Global, Bangalore, India; ^2^ Dept. of Radiation Oncology, Healthcare Global, Bangalore, India; ^3^ Medical Oncology, Healthcare Global, Bangalore, India

**Keywords:** metformin, survival analysis, triple negative breast neoplasm, remission, neoadjuvant

## Abstract

**Background:**

Triple negative breast cancer (TNBC) is more prevalent among the younger age group and is the most aggressive form. Women with TNBC phenotype have 19% lower 5-year overall survival and 18% lower disease-free survival than their non-TNBC counterparts (1). Hence, extensive research is essential for the development of treatment modalities with high efficacy. Metformin is a widely used anti-diabetic drug. The research work has potential implications for re-purposing metformin in the clinical management of TNBC.

**Methods:**

The study design includes a phase 2 randomized clinical trial of TNBC, with metformin given for the intervention arm and placebo oral tablet for the non-intervention arm. A total of 418 breast cancer patients are randomized with a parallel assignment model (209 each for the intervention and the control arms). This is an open-label trial where the primary purpose is treatment and adheres to the SPIRIT reporting guidelines. The trial will be initiated during January 2025 and is expected to be completed by May 2027.

**Discussion:**

The clinical benefit, pathological response rate, and radiological response will be measured after 4.5 to 6 months of initiating treatment. The study will assess the recurrence-free survival (at intervals of 6 months) and overall survival at 2 years from treatment initiation. Patients who respond to the intervention are expected to have >50% decrease in the size of the primary tumor without the appearance of new lesions. RECIST v1.1 criteria are used to determine the objective tumor response for target lesions.

## Background

Triple negative breast cancer (TNBC) is more prevalent among the younger age group and is the most aggressive form. Women with TNBC phenotype have 19% lower 5-year overall survival and 18% lower disease-free survival than their non-TNBC counterparts (1). Among the Indian population, the high incidence of TNBC can be attributed to lifestyle, obesity, deprived socio-economic status, family history, high mitotic indices, and BRCA1 mutation (2). The treatment of TNBC is a challenge due to the lack of targeted therapies. Hence, extensive research is essential for the development of treatment modalities with high efficacy. This research work has potential implications for the clinical management of TNBC.

Metformin is a widely used anti-diabetic drug. Evidence shows the potential benefit of metformin therapy in cancer patients with diabetes. Ongoing research studies assess the anti-tumor effect of metformin in the early stage of breast cancer (BC). Animal models have shown the enhancing effect of metformin on anti-tumor immunity. The anticancer mechanism of action of metformin is attributed to mTOR (mammalian target of rapamycin) inhibition (3). There is certain evidence on its immunomodulatory effects on cancer cells and the impact on markers of tumor proliferation (3). Our study investigates the immune effect of metformin in BC patients treated with preoperative chemotherapy. The epidemiological data will substantiate its potential use in the treatment of cancer and further the evidence to ascertain the possible benefits. Metformin can act on cancer cells either as an immunotherapy agent or a radiation sensitizer or as a direct anti-proliferative agent (4). Evidence on its dose-limiting toxicity shows that metformin will be a tolerable addition to the chemotherapy regimen (4).

Evidence from pre-clinical and retrospective epidemiological studies (3) indicates the anti-cancer properties of metformin. Barakat et al. (5) report a preclinical study which demonstrates the effectiveness of metformin and doxorubicin combination in reducing the tumor size and preventing recurrence compared with either drug when used alone among BC xenograft mouse model. The evidence (5) also shows the synergistic effect of the combination of metformin and paclitaxel in signaling AMPK, which increases the downregulation of the mTOR pathway, when compared with either drug used alone. The immune effect of metformin has been delineated by Chae YK et al. (4) as inhibition of immune exhaustion of CD8+ TILs, reduction of their apoptosis, and a shifting of the phenotype. This shift includes repositioning of TILs expressing exhaustion markers (PD1, negative; Tim3, positive) from central memory T cells to effector memory T cells.

Chae YK et al. (4) report the reduction in initiation of translation in MCF-7 breast cancer cells due to the use of metformin as well as the inhibition of mTOR and reduced phosphorylation of S6 kinase, ribosomal protein S6, and eIF4E binding protein. The pathways by which metformin inhibits the activation of mTOR, independent of AMPK pathway, include escalating the expression of REDD1 by a p53-mediated inhibition when tested on prostate cancer cells and through Ras-related GTPase as well as TSC1/2. Metformin attenuates the growth of cancer stem cells by impeding the Warburg effect. Moreover, it improves the oxygenation ability of cells and decreases the consumption of oxygen in the mitochondria, thus suppressing the accumulation of hypoxia-induced HIF-1α. These effects form the basis of its anti-cancer activity.

### Epidemiology

#### Global prevalence

As per the GLOBOCAN 2020 report, the age-standardized incidence rate of breast cancer globally is 47.8 per 100,000, and the age-standardized mortality rate is 13.6 per 100,000. For the rest of the world excluding India, the age-standardized incidence rate of breast cancer is 27.5 per 100,000, and the age-standardized mortality rate is 13.2 per 100,000 (6).

Globally, female breast cancer has now surpassed lung cancer as the leading cause of cancer incidence in 2020, with an estimated 2.3 million new cases representing 11.7% of all cancer cases. In addition, it is the fifth leading cause of cancer mortality worldwide, with 685,000 deaths. Among women, breast cancer accounts for one in every four cancer cases and one in six cancer deaths, ranking first for incidence in most countries (6).

#### India prevalence

In India, approximately 100,000 women are diagnosed with breast cancer annually, with a high case fatality ratio of 40%. TNBC occurs in about 20%–43% of all patients with breast cancer. As per the GLOBOCAN 2020 report, the age-standardized incidence rate of breast cancer in India is 25.8 per 100,000, and the age-standardized mortality rate is 13.2 per 100,000 (1).

The prevalence of TNBC among Indian women is highest (27.9%) when compared with other regions of the world: Indonesia (25%), Algeria (20.8%), and Pakistan (18%). With both sexes combined, breast cancer is the most frequently observed cancer (14%) and is one of the leading causes of death (11.1%) in India (1).

## Methods/design

The clinical trial aims to further investigate the re-purposed role of metformin in cancer therapy. This phase 2 trial includes 418 breast cancer patients, randomized with a parallel assignment model (209 each for the intervention and the control arms). Allocation concealment is ensured through sealed envelopes. The calculated sample size for this clinical superiority design is 199 in each arm, and accounting for 5% dropout rate, we shall include 209 patients in each arm of the study. The sample size (7) was calculated considering the overall response rates of 80.56% (intervention arm) and 68.42% (control arm) from the study by Barakat et al. (5).

The formula used for the same was proposed by Kirkwood BR et al. (7):


$$N=\frac{{\left\{u\surd [\pi_{1}-(1-\pi_{1})+\pi_{0}(1-\pi_{0})]+v\surd [2\bar{\pi }(1-\bar{\pi })]\right\}}^{2}}{{\left(\pi_{0}-\pi_{1}\right)}^{2}}$$

$$\bar{\pi }=\frac{\pi 0+\pi 1}{2}$$

**Table:** 

N	Sample size
$\pi_{1}$	proportion of outcome in the intervention group = 0.806
$\pi_{0}$	proportion of outcome in the control group = 0.684
*u*	percentage point of the normal distribution corresponding to the (two-sided) 100% - power (80%), which is 0.84
v	percentage point of the normal distribution corresponding to the (two-sided) significance level = 5%, *v* = 1.96

All of the subjects will be recruited from the host institute. This is an open-label trial where the primary purpose is treatment. The trial will be initiated in June 2025 and is expected to be completed by December 2027. Following a written informed consent, all of the study subjects will be recruited in the initial 6 months of the study and followed for the next 2 years. The clinical benefit, pathological response rate, and radiological response will be measured after 4.5 to 6 months of initiating treatment. The study will estimate the recurrence-free survival (at intervals of 6 months) and overall survival at 2 years from treatment initiation. The recommended items in this clinical trial protocol conform to the SPIRIT guidelines.

The evaluation of ER, PR, and HER2 status expressions in breast cancer will be performed using the American Society of Clinical Oncology/College of American Pathologists (ASCO/CAP) guidelines (8). The clinical staging will be defined according to the AJCC manual, eighth edition (9). All adverse events were graded using the National Cancer Institute Common Terminology Criteria for Adverse Events (NCI-CTCAE), version 4.0.

### Arms and interventions

Early-stage or locally advanced (non-metastatic) TNBC patients placed on standard therapy are recruited for the study. They are placed on standard therapy as recommended by the institute’s multi-disciplinary team. Patients treated with either AC-T (adriamycin, cyclophosphamide, and paclitaxel) or AC (adriamycin and cyclophosphamide) or GC (gemcitabine and carboplatin) or TAC (docetaxel (taxotere, adriamycin, and cyclophosphamide) will be eligible for randomization.

A baseline assessment of the following tumor markers (via a biopsy) and serum markers will be done:

- estrogen receptor, progesterone receptor, human epidermal growth factor-2 receptor, Ki-67
- tumor-infiltrating lymphocyte (TIL) analysis: CD4(+), CD8(+), FOXP3, CD25(+)
- programmed death ligand (PDL1)
- androgen receptor
- microvascular density, AMPK/mTOR pathway
- granzyme B, perforin
- insulin-like growth factor (IGF-1, IGF-2)
- carcinoembryonic antigen (CEA), cCancer antigen 15.3 (CA 15.3), CYFRA 21-1
- lactate dehydrogenase (LDH), alkaline phosphatase (ALP)

Radiological investigation: This is a baseline assessment of the tumor size via a FDG PET-CT (fluorodeoxyglucose positron emission tomography) scan. As shown in **Table 1**, each of these medications (metformin or placebo) will be provided along with standard therapy for the duration of chemotherapy (usually for 18 weeks). As per the Institutional guidelines, post-chemotherapy the patients will be subjected to surgery and/or radiotherapy given the clinical indications. Patients manifesting with gastro-intestinal symptoms of metformin intolerance are supplemented with Probiotics and dose optimization. Patients with severe GI symptoms are discontinued from the trial. 

**Table 1:** Arms of the intervention study.

Sl no.	Arm	Intervention
1	Metformin group	Oral metformin: initial dose of 500–850 mg/day increased to a maximum daily dose of 2,550 mg in three divided doses
2	Placebo group	Placebo drug three times/day

**Table 2:** GANTT chart of the events.

Sl no.	Month and year	Activity	Remarks
1	Jun 2025 to Oct 2025	Recruitment of subjects and study initiation	Subjects recruited and randomized to respective arms
2	Nov 2025 to Apr 2026	Measuring the pathological response rate and radiological response	4.5 to 6 months after treatment initiation
3	Jun 2026 to Oct 2026	Measuring the recurrence-free survival	At intervals of 0.5 year from completion of first-line treatment
4	Jun 2027 to Oct 2027	Measuring the overall survival	2 years from treatment initiation
5	Nov 2027 to Dec 2027	Data analysis and drafting of the manuscript	Submission for publication

**Table 2** depicts the timeline of planned activities, with the recruitment of subjects scheduled to start from Jun 2025 and completion of the follow-up scheduled for Dec 2027.

### Outcome measures

#### Primary: (time frame is 4.5 to 6 months from start of therapy)

The metformin therapy is started preoperatively, and the serum biomarkers (tumor/non-tumor) are again assessed 4.5 to 6 months after standard (completion of chemotherapy) and metformin treatment. The primary aim is to assess the clinical benefit and pathological tumor response in the intervention arm. The primary breast lesion and the axillary lymph nodes will be assessed by physical examination and FDG PET-CT scan prior to the first chemotherapy cycle. A measurable tumor lesion includes a minimum size of 10 mm by PET-CT scan in the longest diameter. A pathologically enlarged lymph node must be >15 mm in the short axis by PET-CT scan (10). The clinical response was measured by the change in the diameter of the target lesions from baseline.

In addition, a midline PET-CT scan will be done at the 12th week. The pathologic complete response (pCR) post-surgery will determine the further assessment of tumor markers in the patient. For patients who do not have pCR, the specimen is assessed for all the tumor markers done during the baseline (including the serum tumor and non-tumor markers as mentioned earlier). The difference in these markers will be assessed in the analysis. Clinico-radiological response includes an endline FDG PET-CT scan assessment of the tumor size after completion of chemotherapy. Patients who respond to the intervention are expected to have >50% decrease in the size of primary tumor without appearance of new lesions. This radiological investigation will be done before posting the patient for surgery.

Clinical response is categorized into four categories as per RECIST v1.1 criteria (10) (with reference to the baseline tumor size), which is used to determine the objective tumor response for target lesions:

- Complete response (CR): This is the disappearance of all target lesions. Any pathological lymph nodes (whether target or non-target) must have reduction in the short axis to<10 mm.
- Partial response (PR): This is at least 30% decrease in the sum of diameters of the target lesions, taking as reference the baseline sum diameters.
- Progressive disease (PD): This is at least 20% increase in the sum of the diameters of the target lesions, taking as reference the baseline sum. In addition, the sum must also demonstrate an absolute increase of at least 5 mm.
- Stable disease (SD): This is tumor response with neither sufficient shrinkage to qualify for PR nor sufficient increase to qualify for PD, taking as reference the baseline sum of diameters.

#### Secondary: (time frame is post-surgery or completion of chemotherapy)

Recurrence-free survival will be assessed at an interval of every 6 months for a duration of 2 years.

Overall survival at 2 years: Patients will be followed-up every year for a duration of 2 years, post-surgery, or upon completion of chemotherapy. This will be done to assess the event/recurrence-free survival (RFS) and overall survival (OS). This assessment includes whole-body FDG PET-CT scan and biomarkers such as circulating tumor cells and cell-free DNA.

The association of clinical and demographic factors with outcomes including pCR and RFS or overall survival (OS) will be determined by the Kaplan–Meier product limit method and multivariate Cox proportional hazard models. Hazard ratio (HR) with 95% confidence intervals (CI) will be measured.

### Eligibility criteria

The study will include adult female patients in the age group 18 to 65 years. This study does not accept healthy volunteers.

#### Inclusion criteria

- Unilateral or bilateral primary breast carcinoma, confirmed histologically by core needle biopsy
- Candidate for neoadjuvant chemotherapy as per the hospital’s protocol
- Clinically measurable tumor (≥1.5 cm) if the node is negative (regardless of size if the node is positive)
- Non-PDL1 and non-BRCA 1/2 patients
- Eastern Cooperative Oncology Group Performance Status (ECOG PS) 0–1
- No evidence of distant metastasis
- Normal renal and liver functions
- Multifocal or multicentric lesions when found triple negative by biopsy; however, the largest lesion will be the target

#### Exclusion criteria

- Metastatic breast cancer patients
- Prior cancer chemotherapy
- Prior radiation therapy for breast cancer
- Pregnant or lactating women or unwilling to practice birth control during study participation
- Patients with hepatic impairment
- Patients with renal impairment
- Diabetics who are being treated with metformin
- History of allergic reactions to metformin
- Patients unwilling to participate or withdrawing from the trial
- Psychological/mental impairment

## Discussion

### Implementation science framework of the trial)

(11) Toward improving the implementation of the trial, relevant strategies will be conceptualized to design the interventions. These strategies are linked to determinants which assist in identifying the facilitators and barriers for the success of a trial. In the context of this trial, implementation interventions based on a theoretical framework score above generic strategies which fail to address the root cause of trial problems. The initial identification of root causes entitles the optimization of an effective design and targeting of interventions.

The clinical trial implementation outcomes conform to the proctor’s implementation outcomes framework (IOF) which informs external validity (e.g., acceptability) and internal validity (reproducibility). The context and determinants specific to the implementation of the clinical trial will be defined using the Consolidated Framework for Implementation Research (CFIR). The implementation of interventions will be guided by mapping contextual determinants to the possible Expert Recommendations for Implementing Change (ERIC) strategies (12). In order to facilitate the intervention-related specifications and its reproducibility and testable causal mechanisms on implementation and clinical trial outcomes, a rigorous tool such as implementation research logic model (IRLM) will be used.

### Collaborating institutions

Health Care Global (HCG) is the largest private cancer care provider in India. It has a network of 22 comprehensive cancer centers in India and abroad. Each center is provided with a business system, management expertise, and capital resources to bring patient-focused, state-of-the-art cancer care. The hub and spoke model has helped to create an integrated approach to cancer care. Annually, >120,000 patients are treated through our centers. The HCG foundation also enables need-based patients through concessions and waiver of hospitalization costs. Since its inception in December 2006, around 1,500 financially deserving patients have derived benefit from this noble cause.

Radiological investigations will be done at HCG, Bangalore, and immunohistochemistry investigations will be done at Triesta Life Sciences, HCG.

Triesta Sciences is a unit of HCG and is the one point, state-of-the-art solution for cancer diagnostics, genomics (next-generation-sequencing-based diagnostics) biomarker and translational research, laboratory services, and clinical research services.

**Table 3:** Summary of the project budget.

Sl no.	Category	Duration (months)	Unit cost (INR)	Total cost (INR)
1	Direct costs			
	Personnel salaries and wages^a^			
1a	Principal investigator (from HCG)	36	30,000	1,080,000
1b	Co-PI 1 (from HCG)	36	30,000	1,080,000
1c	Co-PI 2 (from iCrest)	36	30,000	1,080,000
1d	Coordinator	36	15,000	540,000
1e	Statistician	2	25,000	50,000
2	Indirect costs			
2a	Radiology investigation^b^		20,000	8,360,000
2b	Pathology investigation^c^		35,000	14,630,000
2c	Supplies and materials^d^			400,000
3	Sub-total			27,220,000
4	Institutional overheads			
	HCG (10% of sub-total)			2,722,000
5	HCG ethics committee			30,000
6	Total^e^			29,972,000

a Project personnel costs include conducting trial, data gathering, curation, and analysis.

b FDG PET-CT scan at midline (as baseline and endline are borne by patients as part of treatment).

c Tumor markers, serum markers (tumor, non-tumor) including blood immune profiling at midline for patients (as baseline and endline investigations are borne by patients as part of treatment).

d Drug (metformin), 3-in-1 printer, dedicated laptop, and data analysis software.

The project budget is listed in **Table 3**. This includes direct costs which are the salaries of human resources and indirect costs which are the logistics and investigations.

### Data collection

The electronic data capture of the tool REDCap (Research Electronic Data Capture) will be used to collect and manage the study data. This web-based application provides an interface for validated data entry, tracking data manipulation, automated export procedures to common statistical packages, and avenues for importing data from external sources.
