# Correction: Neoadjuvant chemotherapy with or without metformin in early-stage or locally advanced (non-metastatic) triple negative breast cancer—an open-label phase 2 randomized controlled trial

**DOI:** 10.3389/fonc.2025.1658958

**Published:** 2025-08-01

**Authors:** Vinod K. Ramani, Ajaikumar Basavalinga Sadashivaiah, Radheshyam Naik

**Affiliations:** ^1^ Preventive Oncology, Healthcare Global, Bangalore, India; ^2^ Radiation Oncology, Healthcare Global, Bangalore, India; ^3^ Medical Oncology, Healthcare Global, Bangalore, India

**Keywords:** metformin, survival analysis, triple negative breast neoplasm, remission, neoadjuvant

In the published article, there was an error in the affiliation of the first author. Instead of “Preventive Oncology, Sammprada Cancer Hospital, Bangalore, India”, it should be “Preventive Oncology, Healthcare Global, Bangalore, India”.

In the published article, there was an error in the affiliation of the third author. Instead of “Preventive Oncology, Sammprada Cancer Hospital, Bangalore, India”, it should be “Medical Oncology, Healthcare Global, Bangalore, India”.

The corresponding author email was erroneously given as: vinodramani77@gmail.com. The correct email address is: drvinod.r@hcgel.com.

The original version of this article has been updated.

